# Multi-Attribute Decision Making: Parametric Optimization and Modeling of the FDM Manufacturing Process Using PLA/Wood Biocomposites

**DOI:** 10.3390/ma17040924

**Published:** 2024-02-17

**Authors:** Alexandra Morvayová, Nicola Contuzzi, Laura Fabbiano, Giuseppe Casalino

**Affiliations:** Dipartimento di Meccanica, Matematica e Management, Polytechnic University of Bari, Via Orabona 4, 70125 Bari, Italy; alexandra.morvayova@poliba.it (A.M.); nicola.contuzzi@poliba.it (N.C.); laura.fabbiano@poliba.it (L.F.)

**Keywords:** biocomposite, PLA/wood, FDM, optimization process, Grey Relational Analysis, Taguchi orthogonal array

## Abstract

The low carbon footprint, biodegradability, interesting mechanical properties, and relatively low price are considered some of the reasons for the increased interest in polylactic acid-based (PLA-based) filaments supplied with natural fillers. However, it is essential to recognize that incorporating natural fillers into virgin PLA significantly impacts the printability of the resulting blends. The complex inter-relationship between process, structure, and properties in the context of fused deposition modeling (FDM)-manufactured biocomposites is still not fully understood, which thus often results in decreased reliability of this technology in the context of biocomposites, decreased accuracy, and the increased presence of defects in the manufactured biocomposite samples. In light of these considerations, this study aims to identify the optimal processing parameters for the FDM manufacturing process involving wood-filled PLA biocomposites. This study presents an optimization approach consisting of Grey Relational Analysis in conjunction with the Taguchi orthogonal array. The optimization process has identified the combination of a scanning speed of 70 mm/s, a layer height of 0.1 mm, and a printing temperature of 220 °C as the most optimal, resulting in the highly satisfactory combination of good dimensional accuracy (Dx = 20.115 mm, Dy = 20.556 mm, and Dz = 20.220 mm) and low presence of voids (1.673%). The experimentally determined Grey Relational Grade of the specimen manufactured with the optimized set of process parameters (0.782) was in good agreement with the predicted value (0. 754), substantiating the validity of the optimization process. Additionally, the research compared the efficacy of optimization between the integrated multiparametric method and the conventional monoparametric strategy. The multiparametric method, which combines Grey Relational Analysis with the Taguchi orthogonal array, exhibited superior performance. Although the monoparametric optimization strategy yielded specimens with favorable values for the targeted properties, the analysis of the remaining characteristics uncovered unsatisfactory results. This highlights the potential drawbacks of relying on a singular optimization approach.

## 1. Introduction

In recent years, there has been a discernible concentration within the expansive research community on the application of biocomposites across diverse domains. In both research and industrial contexts, biocomposites encompass composite materials composed of a matrix and reinforcements, sourced predominantly from biological origins, exemplified by combinations like polylactic acid (PLA) with cellulose, wooden fibers, cork, and hemp [1,2,3].

The recognized advantages of biocomposites encompass heightened mechanical strength relative to unadorned biopolymers, an advantageous price–quality ratio, and a renewable provenance, with the natural fillers often derived from various industrial processes or waste by-products. Noteworthy is the facile disposal or recyclability of both the matrix and fillers post their service cycle, devoid of deleterious impacts on the environment. Facilitated by their cost-effectiveness, biocomposites have overcome their limitations to specialized applications in science and research, finding seamless integration into commonplace objects and applications where their ecological attributes can yield consequential environmental benefits [4,5].

Considering the current escalation of greenhouse gas levels and the accelerating pace of global warming, it is reasonable to posit that novel, globally applicable ecological solutions, including the adoption of alternative materials like biocomposites, may play a key role in successfully fulfilling the United Nations Sustainable Development Goals [6,7].

In recent years, additive manufacturing has emerged as an innovative and virtually waste-free technology, challenging conventional manufacturing paradigms. Given the polymeric nature of biocomposites, Fused Deposition Modelling (FDM) presents a promising technology for their successful processing [8].

However, it is important to recognize that the introduction of natural reinforcements to virgin biopolymers can have profound effects on the biocomposites’ printability. Variations in the densities of matrix and fillers may induce turbulent flow in the molten filament, leading to the clustering of filler particles and nozzle clogging. It was determined that nozzle clogging is mainly affected by fiber aspect ratio and orientation. Therefore, these parameters must be controlled [9,10,11]. Moreover, the hydrophilic properties of certain natural fibers used as fillers can adversely impact the dimensional stability of manufactured components, decrease filament durability, and ultimately influence the mechanical properties of the fabricated pieces [12,13].

To attain biocomposite specimens of superior quality with precise geometrical accuracy, an optimal processing window for FDM must be identified. Nevertheless, the FDM manufacturing of biocomposites is a polyparametric intricate problem, and the underlying process–structure–property relationships remain elusive. Consequently, the current reliability of FDM manufacturing for biocomposites is somewhat constrained, posing a potential impediment to its widespread industrial applications. Addressing these challenges is crucial for unlocking the full potential of FDM in the manufacturing of biocomposites on a global scale [14,15].

Several attempts to improve the properties of FDM-manufactured PLA/wood biocomposites have been reported in the literature. Csizmadia et al. [16] aimed to increase the tensile strength of the biocomposite by chemical impregnation of the wood fibers. While this approach was proved to be successful in both increasing the mechanical properties of the composites and decreasing the water absorption of the final blends, the additive of various impregnators, such as phenolic resins might substantially influence their biodegradability and thus can be no longer suitable for the applications where biodegradability is necessary. Similarly, Trinh et al. [17] studied the possibility of improving the properties of the PLA/wood biocomposites by grafting the maleic anhydride onto the virgin PLA. It was determined that the inclusion of the maleic anhydride can significantly suppress the thickness swelling by increasing the water resistance of the blends when compared to the standard PLA/wood biocomposites. Singh et al. [18] have successfully integrated the CRITIC–MABAC-Based Decision-Making algorithm in optimizing the chemical composition of wood/rice husk-waste-filled PLA biocomposites to achieve the components with optimal physical and mechanical properties. The authors determined the blends of PLA with a 7.5% wood content as the optimal biocomposite composition. When considering the application of FDM in processing the PLA/wood blends, several researchers [19,20,21] have described that the final properties of the FDM-manufactured composites highly depend on the applied process parameters.

Some of the key parameters of the FDM process influencing both the dynamics of the process itself and the overall properties of the manufactured components include printing angle, layer thickness, fill rate, nozzle temperature, building direction, infill percentage, and the eventual application of the postprocessing techniques. In general, the manufacturing process’s dynamics affect the deposited material’s temperature, its flowability, and the adhesion of the newly deposited layer to the prevenient one. The parameters mentioned above have been found to affect tensile strength, modulus of elasticity, ultimate strength, and elongation at break. Notably, these mechanical properties are affected by the interlayer bonding between deposited layers and the potential presence of defects and voids. Additionally, factors such as surface roughness, mechanical strength, and dimensional accuracy are also influenced by parameters like layer thickness, infill density, air gap, and raster width. Therefore, careful consideration and optimization of these parameters are essential for achieving the desired quality and properties in FDM-manufactured parts [22,23,24].

Despite the intensive ongoing research, the unambiguous determination of the optimal set of the FDM process parameters, resulting in the biocomposite components of outstanding quality, remains a challenge.

In response to the challenges associated with the limited reliability of FDM processing of biocomposites, this study presents the application of Grey Relational Analysis (GRA) methodology, coupled with the Taguchi orthogonal array for the systematic determination of the optimal FDM processing parameters. The objective is to achieve superior geometrical accuracy and close-to-full density in the FDM-manufactured cubes composed of Polylactic acid (PLA)-based biocomposites incorporating wooden fibers. Response parameters, specifically porosity, and dimensions in the x-, y, and z-directions, were selected based on the existing literature, which underscores the prevalence of porosity as a common concern in FDM manufacturing. Porosity significantly impacts the mechanical properties of fabricated components. Moreover, distortions emerge as a prominent risk when processing biocomposites containing hydrophilic wooden fillers. This study aims to address these challenges through the systematic optimization of FDM processing parameters, with a focus on achieving enhanced geometrical precision and density in PLA-based biocomposites. The optimization performance of this multiparametric approach is subsequently compared to the standard Taguchi optimization process, aiming to achieve the desired properties of the manufactured biocomposites one at a time in four different optimization processes.

## 2. Materials and Methods

### 2.1. Materials and Manufacturing

The biocomposite applied in this study to manufacture the specimens consisted of 80% PLA and 20% filler in the form of wooden flour (Sunlu, China). The wood particles loaded into the PLA filament were composed of pine wood. They were of irregular shape, with dimensions between 0.25 and 0.35 mm. The feedstock filament had a diameter of 1.75 mm, with a dimensional accuracy of +/−0.03 mm. The density of the filament, as given by the manufacturer, was 1240 kg/m^3^.

This study used a RepRap (Anet A8, Anet Technology Co., Ltd., Shenzhen, China) FDM printer. Three different printing parameters (scanning speed (SS), layer height (LH), and printing temperature (PT)) and 4 different levels were considered, as demonstrated in Table 1.

No heated support platform was employed. To secure adherence of the specimens to the build plate, a water-soluble glue was applied, forming a thin film on the building platform to prevent detachment of the specimens.

The combination of the process parameters applied to manufacture the specimens was planned according to the Taguchi orthogonal array, to reduce the number of experimental sets. The applied Taguchi orthogonal array design L_16_ has resulted in 16 experimental runs (Table 2). To ensure the causality between the applied processing parameters and the studied properties of the manufactured cubes, the whole experimental procedure was replicated 3 times, resulting in the full number of 48 manufactured and analyzed specimens. The statistical analysis was performed to contradict the statistically significant differences in the populations of the replicates. The tested properties of the analyzed cubes stated in this paper are the average values of all three replicates manufactured with the same processing parameters.

Moreover, in the next phase of the research, additional specimens were manufactured, using the process parameters determined as optimal resulting from the multiparametric and monoparametric optimization processes.

**Figure 1:**
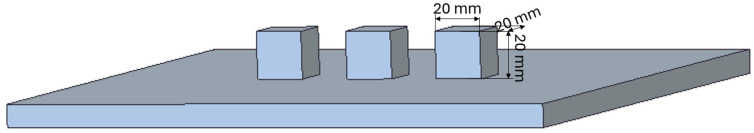
Biocomposite specimens and their position on the building platform.

The specimens were arranged in cubes, each having an optimal edge length of 20 mm. Samples produced with identical process parameters were created simultaneously, centrally positioned on the build plate adjacent to one another (see Figure 1). Thus, the manufacturing process comprised 16 cycles, with each cycle employing a distinct combination of process parameters, and within each cycle, three specimens were produced as replicates.

The manufacturing process involved a raster building strategy with 67° of rotation between the successive layers.

### 2.2. Porosity Testing

**Figure 2:**
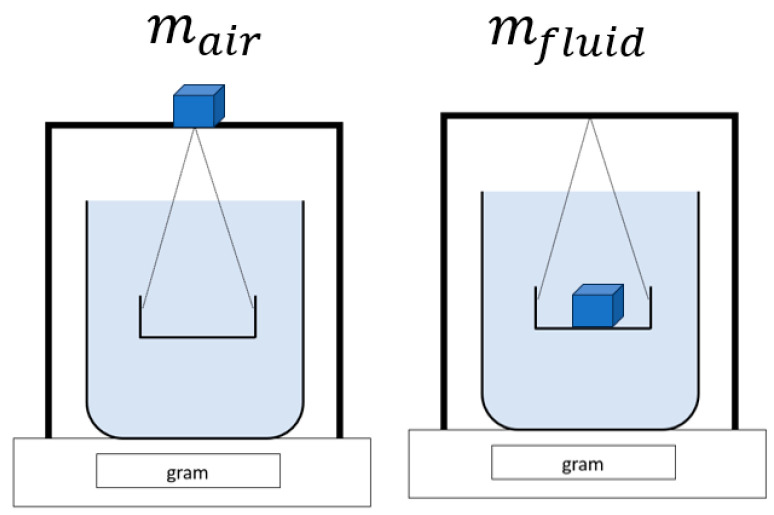
Scheme of Archimedes’ method in determining the porosity of the manufactured biocomposites.

Porosity, or in general presence of cavities is one of the most common defects in FDM and its impact on the mechanical properties of the manufactured components is unquestionable. Therefore, after completing the building process, all the manufactured specimens were subjected to relative density testing according to Archimedes’ method, as seen in Figure 2. The porosity of the sample was further calculated as in Equation (2).

(1) $$\rho_{specimen}=\rho_{fluid}\frac{m_{air}}{\left(m_{air}-m_{fluid}\right)}$$

(2) $$Porosity=100-(\frac{\rho_{specimen}}{\rho_{AlSi10Mg}}\;\cdot \;100)$$

### 2.3. Dimensions Testing

**Figure 3:**
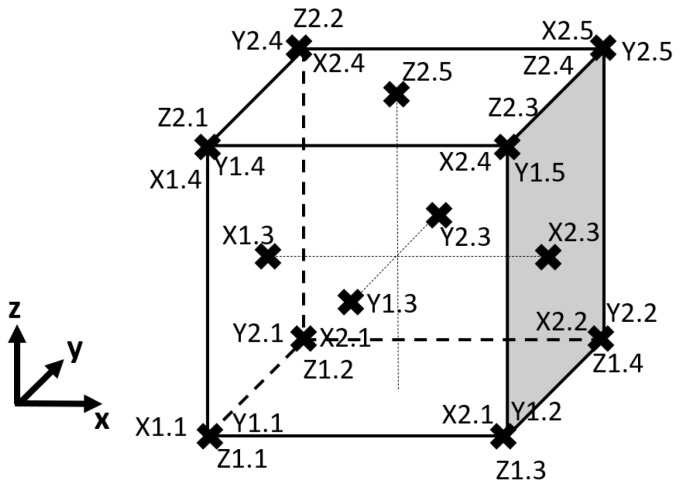
Distribution of the measuring points on the FDM-manufactured components.

The dimensions of the manufactured samples were investigated as the distances between the parallel faces of the cubes. The measurements were assessed in 5 measuring points distributed at each plane. Each assessed face contained 5 points, where 1 point was oriented in the middle of the face of the cube, and the other 4 were symmetrically distributed on the cubes’ corners, as shown in Figure 3.

## 3. Optimization Methodology

### 3.1. The Multiparametric Optimization Algorithm

Grey Relational Analysis (GRA) was employed to optimize the process parameters of the Fused Deposition Modeling technique, addressing the multifaceted challenges associated with achieving optimal geometrical accuracy and minimal porosity in FDM-manufactured cubes fabricated from wood-filled biocomposites. This analytical approach is particularly well suited for scenarios involving multiperformance issues, where optimization proves challenging due to factors such as limited experimental data. In contrast to conventional Taguchi design of experiment (DOE) methodologies, GRA demonstrates applicability in the context of multiresponse optimizations, accommodating diverse performance characteristics [25,26].

Notably, GRA possesses the unique capability to address optimization challenges when responses exhibit variations in scale. This advantageous feature enables the method to transcend limitations associated with standard DOE methodologies. The normalization process employed in GRA facilitates optimizing responses with differing scales. By transforming experimental data into normalized values within the range of 0–1, the dependency on specific units is eliminated, enhancing the versatility and generalizability of the optimization process. The normalization process was applied to decrease the input variability, while the smaller-to-better approach was adopted (Equation (3)) [27]:

(3) $$x_{i}^{*}\left(k\right)=\frac{\max x_{i}\left(k\right)-x_{i}(k)}{\max x_{i}\left(k\right)-\min x_{i}(k)}$$

where $x_{i}^{*}\left(k\right)$ denotes the normalized value, $\max x_{i}\left(k\right)$ and $\min x_{i}(k)$ denotes the maximum and minimum value of the response parameter in the original state, respectively. Notably, the smaller-to-better approach was utilized in conformity with the goal of the optimization process to suppress the porosity. Following our previous research works, we have expected the FDM manufacturing process to result in oversized dimensions of the manufactured biocomposites. Since we have aimed to suppress such distortions within the optimization process, the smaller-to-better approach was again adopted.

Furthermore, the GRA methodology proceeds with the calculation of the Grey Relational Coefficient $\varepsilon_{i}\left(k\right)$, which is expressed as in Equations (4) and (5) [28]:

(4) $$incrementincrement\varepsilon_{i}\left(k\right)=\frac{{∆}_{min}+\varepsilon {∆}_{max}}{{∆}_{oi}\left(k\right)+\varepsilon {∆}_{max}}$$

(5) $${∆}_{oi}\left(k\right)=\left\|x_{o}\left(k\right)-x_{i}(k)\right\|$$

where ${∆}_{min}$ and ${∆}_{max}$ refer to the minimum and maximum value of the absolute differences of all analyzed sequences and *ε* is the identification coefficient, commonly chosen as 0.5. ${∆}_{oi}\;(k)$ is the deviation sequence of the reference sequence $x_{o}\left(k\right)$ and the comparability sequence $x_{i}(k)$.

Subsequently, the Grey Relational Grade (γ), presenting the level of correlation between the reference sequence and the comparability sequence and is the overall representative of all the quality characteristics is calculated as in Equation (6). Notably, by determining the Grey Relational Grade, the multiresponse problem is converted into a single response optimization problem [29,30].

(6) $$\gamma_{i}=\frac{1}{n}\sum_{k=1}^{n}\varepsilon_{i}(k)$$

where *n* denotes the number of responses, in this case, 4, notably, the highest relational grade represents the values of process parameters that are closest to the optimal combination. Thereafter, the available experimental runs containing different combinations of FDM process parameters are ranked according to their Grey Relational Grade.

The graded and ranked parameters represent a single response optimization problem, that is solved through standard Taguchi DOE, where the means of Grey Relational Grade for each factor at each level has been calculated. For each factor, the level with the highest means of Grey Relational Grade was identified. The identified combination of the FDM process parameters represents the optimal setting window with which the lowest porosity and highest dimensional accuracy can be achieved [31].

Furthermore, the ANOVA analysis was performed to evaluate the statistical significance of all the FDM process parameters on the Grey Relational Grade. Notably, the 95% level of significance and 3 degrees of freedom (DF) for each one of the evaluated process parameters were chosen in the statistical analysis. The ANOVA evaluated the mean square (MS), sum of squares (SS), F-ratio, and *p*-value for both scanning speed, layer height, and printing temperature.

The results of the optimization are experimentally verified through the additional experimental run applying the optimal set of process parameters. The optimal experimental specimen is further subjected to porosity and dimension measurements, as it is described in Section 2.2 and Section 2.3. The Grey Relational Grade of the experimental specimen is compared to the predicted optimized value. The predicted Grey Relational Grade for the optimal set of process parameters can be calculated as follows (Equation (7)).

(7) $$\hat{\gamma }=\gamma_{m}+\sum_{i=1}^{o}\left({\bar{\gamma }}_{i}-\gamma_{m}\right)$$

where $\hat{\gamma }$ represents the predicted value of the Grey Relational Grade of the optimized set of process parameters, $\gamma_{m}$ is the mean value of the Grey Relational Grade of all the tested parameters, o is the number of the process parameters involved in the optimization process, and ${\bar{\gamma }}_{i}$ is the mean Grey Relational Grade of each process parameter at the optimal level [32,33].

### 3.2. The Monoparametric Optimization Algorithm

Additionally, the present study adopts the standard monoparametric Taguchi orthogonal array and compares its optimization performance with the multiparametric approach. In a monoparametric approach, the process parameters are optimized to achieve the desired properties of the biocomposite cubes one at a time, in 4 different cycles. Unlike in the previous section, the assigned lowest values of the means of the inspected characteristics are considered the most optimal. Therefore, the smaller-to-better approach is adopted, and the optimal levels of all the examined process parameters are then chosen accordingly. The effects of the FDM process parameters on the examined characteristics of the biocomposite cubes are evaluated through ANOVA. The experimental results of the specimens manufactured under the optimized sets of process parameters are then compared with each other, as well as with the ones manufactured under the set of process parameters optimized with the multiparametric approach.

## 4. Results and Discussion

### 4.1. Experimental Results

Table 3 lists the experimentally assessed dimensions and porosity of the 16 biocomposite cubes manufactured with different combinations of printing speed, layer height, and printing temperature.

Upon closer examination of the evaluated features, it was apparent that the dimensions and porosity of biocomposites, produced under distinct process parameters, exhibited considerable variability. Significantly, only oversized dimensions of the fabricated cubes have been documented. Various authors propose that this discrepancy may originate from slicer settings, the pronounced heat expansion coefficient of polylactic acid (PLA), and the hydrophilic nature of the wooden additives [34,35,36].

**Figure 4:**
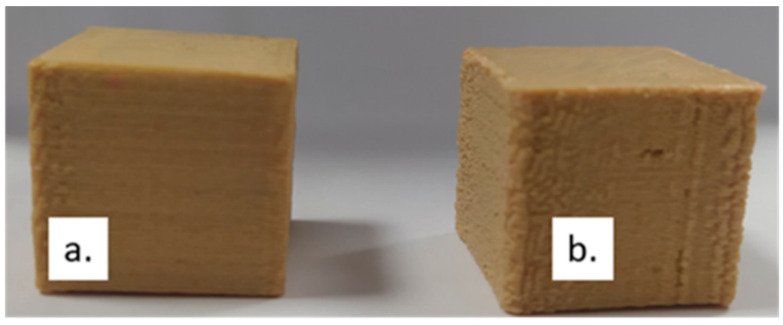
Experimental specimens manufactured with (**a**) SS = 110 mm/s, LH = 0.10 mm, and PT = 210 °C; (**b**) SS = 110 mm/s, LH = 0.25 mm, and PT = 200 °C.

The specimens manufactured under different processing conditions are compared in Figure 4. While in Figure 4a, a specimen with relatively even edges was demonstrated, Figure 4b showed a specimen with significantly distorted edges, holes, and cavities of different ranges. On closer inspection of all the examined specimens, it has been revealed that the increase in the layer height generally results in specimens with more distortions and more frequent defects. This observation is supported by the literature [37,38].

The presence of cavities and hole-like defects in cubes manufactured with a layer height of 0.25 mm was supported by higher values of porosity identified in these specimens. Conversely, the highest density was observed in cubes manufactured with the lowest layer height. The effects of the remaining process parameters were much more subtle and could not have been unambiguously generalized. The effect of the processing parameters on distortions, mechanical properties, and defect formation is currently progressively studied [19,20,21].

The porosity of the manufactured biocomposites ranged from 1.53% to 12.87%. Contemporary literature characterizes porosity in FDM-manufactured components as inherent byproducts of the manufacturing process, subject to influence by diverse factors such as filament extrusion processes, deposition path algorithms, and the utilization of thermally expandable second phases in filaments. Noteworthy were the elevated porosity values observed in specimens 11, 12, and 16, indicating the presence of larger-scale defects, such as cavities or substantial voids.

It is imperative to acknowledge that both distortions and porosity are undesirable attributes of FDM-manufactured biocomposites, necessitating urgent mitigation efforts. Consequently, there is a pressing need to address and suppress these adverse features to enhance the overall quality and performance of fabricated biocomposite materials [14,36].

### 4.2. Multiparametric Optimization Process

#### 4.2.1. Grey Relational Analysis Results

The optimization process first normalized the experimental values and then calculated the Grey Relational Coefficients. Subsequently, each combination of FDM process parameters was given a Grey Relational Grade, according to which the examined combinations were ranked. The data obtained throughout the optimization process are listed in Table 4. Since all the measured dimensions of the biocomposite cubes manifested oversized values (Table 3), we were able to apply the smaller-to-better approach, as explained in Section 3.

#### 4.2.2. Results of GRA-Coupled Taguchi Optimization Process in Multiparametric Problem

According to the ranking presented in Table 4, it becomes obvious that the combination of process parameters number 13, consisting of a scanning speed of 130 mm/s, a layer height of 0.1 mm, and a printing temperature of 220 °C results in the most appealing combination of geometrical accuracy and porosity out of all the manufactured biocomposite cubes.

The coupled Taguchi optimization process (Table 5) has revealed that the most optimal combination of the FDM process parameters consists of a scanning speed of 70 mm/s, a layer height of 0.1 mm, and a printing temperature of 220 °C when comparing the means of Grey Relational Grades of each factor at each level. Noteworthy, the optimal combination of process parameters is in bold font and the exact values of process parameters are in Table 5 replaced by the number of levels in ascending order.

**Figure 5:**
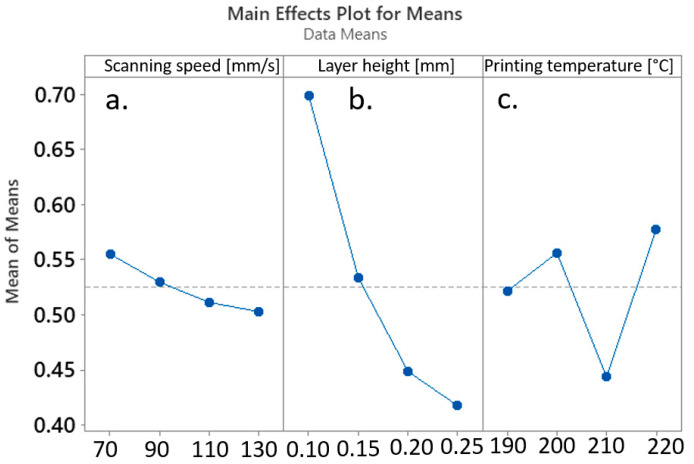
Main effects Plot for Means of Grey Relational Grade affected by (**a**) scanning speed, (**b**) layer height, and (**c**) printing temperature.

The main effect plots for means of Grey Relational Grades and their relationship with the values of the applied process parameters have determined the inverse proportion between the mean Grey Relational Grade and the applied scanning speed and layer height (Figure 5a,b), where, e.g., high values of scanning speed and layer height resulted in low means of Grey Relational Grade, and vice versa. In contrast, the effect of the printing temperature on the mean Grey Relational Grade seemed to be much more irregular (Figure 5c).

It should be noted that the predicted value of the Grey Relational Grade of the optimized set of process parameters was calculated as 0.782.

The ANOVA analysis has further revealed that only layer height had a statistically significant effect on the Grey Relational Grade, while the two remaining parameters did not affect the Grey Relational Grade in a statistically significant manner (Table 6). Notably, the ANOVA is limited by finding only the linear relationships between the examined process parameters and the Grey Relational Grades, while such relationships, especially in the context of manufacturing of the biocomposite components might be more complex.

### 4.3. Taguchi Optimization of Dx, Dy, Dz, and Porosity as the Monoparametric Problems

#### 4.3.1. x-Dimension Optimization

Table 7 enumerates the means of the x-dimensions, computed for each examined process parameter at every respective level. It is noteworthy that the lowest means of the x-dimension associated with each considered process condition, delineating the optimal levels of the process parameters, have been emphasized using bold font.

**Figure 6:**
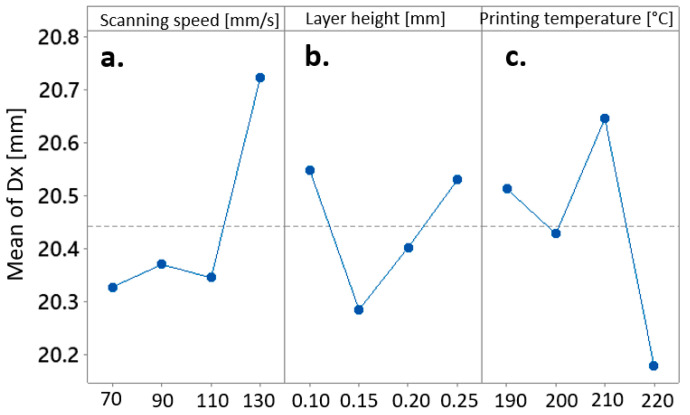
Main effects Plot for Means of the x-dimension affected by (**a**) scanning speed, (**b**) layer height, and (**c**) printing temperature.

In light of optimizing the processing window, the amalgamation of FDM process parameters, encompassing a scanning speed of 70 mm/s, a layer height of 0.15 mm, and a a printing temperature of 220 °C, can be deemed as the most optimal configuration for attaining the utmost precision in the x-dimension of the fabricated cube. The mean effect plots describing the interdependencies between the x-dimension of the produced biocomposite cube and the variables of scanning speed (Figure 6a), layer height (Figure 6b), and printing temperature (Figure 6c) are presented in Figure 5.

Nevertheless, the ANOVA determined that none of the examined process parameters has a statistically significant effect on the x-dimension of the cubic specimen (Table 8).

#### 4.3.2. y-Dimension Optimization

Table 9 lists the means of the y-dimension calculated for each examined process parameter at each level. In accordance with the oversized y-dimensions observed in all the manufactured cubes, the smaller-to-better approach was considered. Therefore, the 130 mm/s, 0.1 mm, and 190 °C were determined as the optimal levels of scanning speed, layer height, and printing temperature, respectively, in achieving the cubes with the most accurate y-dimensions.

**Figure 7:**
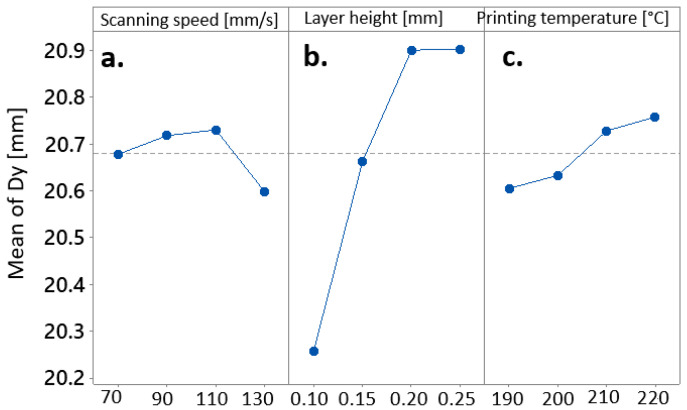
Main effects Plot for Means of the y-dimension affected by (**a**) scanning speed, (**b**) layer height, and (**c**) printing temperature.

As evident from the main effects plots in Figure 6, the sequential increase in layer height from 0.1 mm to 0.2 mm resulted in a significant rise in distortion along the y-dimension of the manufactured cubes (Figure 7b). The impact of further increasing the layer height to 0.25 mm appeared to be less pronounced. Notably, the mean y-dimension of the biocomposite cubes seemed to exhibit a direct proportionality to the printing temperature. However, this effect was considerably less significant when compared to the impact of layer height (Figure 7c). In contrast, the relationship between printing speed and the mean y-dimension showed irregular patterns (Figure 7a).

The significance of the effect of the layer height on the y-dimension was confirmed by the ANOVA analysis (Table 10), while the effect of the two remaining process parameters has been determined not to be statistically significant.

#### 4.3.3. z-Dimension Optimization

**Figure 8:**
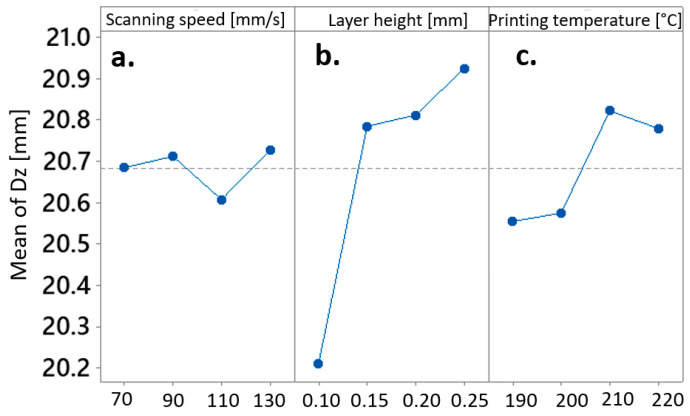
Main effects Plot for Means of the z-dimension affected by (**a**) scanning speed, (**b**) layer height, and (**c**) printing temperature.

The outcomes of the optimization process aimed at minimizing distortion in the z-dimension of the biocomposite cubes are summarized in Table 11. The optimized set of process parameters comprises a scanning speed of 110 mm/s, a layer height of 0.1 mm, and a printing temperature of 190 °C. Notably, the optimized parameters for minimizing distortion in the z-dimension closely resemble those for minimizing distortion in the y-dimension. This similarity suggests that the distortion formation process may be akin in both directions, possibly involving layer height as the primary influencing parameter. This alignment is further supported by the main effects plots (Figure 8), where the curves exhibit a comparable trend to those in Figure 7, and by ANOVA analysis (Table 12), which identified layer height as the process parameter with a statistically significant effect on the resulting z-dimension of the FDM-manufactured biocomposite cube.

#### 4.3.4. Porosity Optimization

Table 13 lists the results of the Taguchi-supported optimization process in achieving the biocomposites with suppressed porosities. The optimized set of process parameters thus included a scanning speed of 70 mm/s, a layer height of 0.1 mm, and a printing temperature of 220 °C. Notably, the identified set of process parameters was the same one that was identified as optimal when solving the multiparametric problem.

**Figure 9:**
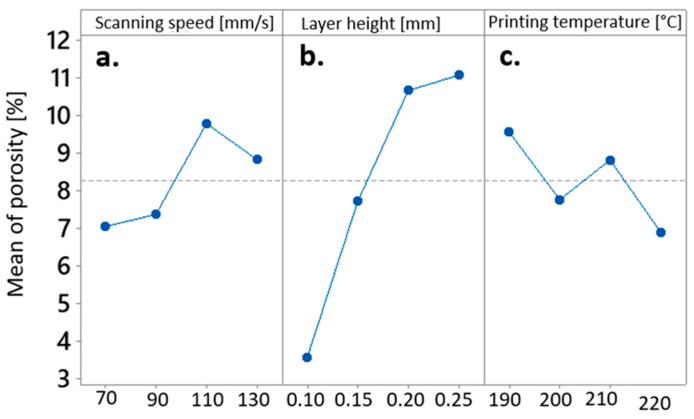
Main effects Plot for Means of porosity affected by (**a**) scanning speed, (**b**) layer height, and (**c**) printing temperature.

The main insights drawn from the main effects plot illustrating the influence of FDM processing parameters on porosity consistently mirror the inverted curves that represent the impact of printing parameters on the Grey Relational Grade within a multiparametric framework. Analogous to the findings expounded in Section 4.3.2 to Section 4.3.3, it was evident that the layer height plays a predominant role in determining porosity outcomes, whereby an increase in layer height corresponded to an augmentation in porosity, and conversely (refer to Figure 9b). In contrast, the effects of printing speed (Figure 9a) and printing temperature (Figure 9c) manifested a more irregular pattern.

Unlike in all the previous sections, the ANOVA has revealed the statistically significant effect of all the FDM process parameters on the resulting porosity in the manufactured biocomposite cubes (Table 14).

### 4.4. Validation of the Optimization Process

#### 4.4.1. Validation of the Multiparametric Optimization Approach

The experimentally assessed dimensions and porosity of the biocomposite cube manufactured through the FDM process with the optimized process parameters (SS = 70 mm/s, LH = 0.1 mm, PT = 220 °C) are listed in Table 15.

The optimized set of process parameters has resulted in biocomposite specimens disposing with a low level of porosity and slightly oversized dimensions.

Table 16 provides the summary of the standardized values of the experimentally assessed dimensions and porosity of the biocomposite cubes manufactured with optimized FDM process parameters, the pertaining Grey Relational Coefficient, and the final value of the Grey Relational Grade.

In this study, it is important to highlight that the Grey Relational Grade (GRG) of the experimental specimen, fabricated using an optimized set of process parameters, was closely aligned with the predicted value (0.782). This congruence underscores the successful validation of the optimization process.

Conversely, the GRG of specimens produced with the initial process parameters ranked first in terms of GRG value, exhibited a marginally higher grade compared to the optimized set. This discrepancy may be attributed to the potentially nonlinear nature of the process-property relationship within the context of FDM processing of biocomposites, as well as the existence of the additional parameters of the manufacturing settings that might significantly influence the characteristics of the final components [39,40].

#### 4.4.2. Monoparametric Optimization Approach Validation

Table 17 compares the experimentally assessed x-, y-, z-dimensions and porosity of the cubes manufactured under the optimized process parameters for each optimization process. Notably, the last set of process parameters was determined to the optimized in both the monoparametric approach optimizing the porosity, and the multiparametric optimization approach. Bolded are the values of the cubes’ properties for which were the optimization processes intended.

The monoparametric approach yielded highly satisfactory values for the targeted properties, demonstrating its efficacy in achieving the intended optimization goals. However, an analysis of additional properties revealed undesired outcomes, suggesting potential limitations of this singular optimization strategy.

Contrastingly, the multiparametric optimization approach, employing Grey Relational Analysis (GRA) in conjunction with the Taguchi orthogonal array, demonstrated significant success. Except for the y-dimension, this approach yielded an overall favorable combination of properties in the manufactured cubes. The resultant property values were comparable to those achieved through monoparametric optimization specifically tailored for individual cube properties.

Our findings highlight the efficiency of the multiparametric approach, emphasizing its utility as a robust tool for determining optimal processing parameters in diverse industrial settings, particularly in the domain of FDM manufacturing with biocomposites. The integration of GRA and Taguchi orthogonal array emerges as a powerful methodology for efficiently navigating the complex parameter space and achieving desirable outcomes across a spectrum of properties, thereby contributing to the advancement of manufacturing processes.

## 5. Conclusions

In this study, the enhancement of geometrical accuracy and the reduction in porosity in FDM-manufactured biocomposites were pursued through the application of Grey Relational Analysis coupled with Taguchi orthogonal array. Furthermore, through a comparative analysis of the optimization performance, this multiparametric approach was assessed against the conventional Taguchi monoparametric optimizations. Several conclusions were drawn:

- The integrated methodology of Grey Relational Analysis (GRA) with the Taguchi orthogonal array demonstrates a capacity to systematically identify and recommend optimal combinations of process parameters more effectively than the conventional monoparametric optimizations.
- The monoparametric optimization approach has resulted in specimens with highly satisfactory values for the targeted properties, making this approach highly efficient for the intended optimization goals. However, an analysis of additional properties revealed undesired outcomes, suggesting potential limitations of this singular optimization strategy.
- Among all the initial experimental combinations of process parameters investigated, the multiparametric optimization process determined that a scanning speed of 130 mm/s, a layer height of 0.1 mm, and a printing temperature of 220 °C yielded the printed biocomposite specimen with the most optimal characteristics (Dx = 20.14 mm, Dy = 20.01 mm, Dz = 20.18 mm, and porosity = 2.44%).
- Importantly, an optimization process identified a new combination of parameters, namely a scanning speed of 70 mm/s, a layer height of 0.1 mm, and a printing temperature of 220 °C, as the most optimal for achieving favorable geometrical accuracy (Dx = 20.115 mm, Dy = 20.556 mm, and Dz = 20.220 mm) and low porosity (1.673%) in the manufactured biocomposites.
- The Grey Relational Grade (GRG) of the experimental specimen, fabricated using an optimized set of process parameters (0.754), was closely aligned with the predicted value (0.782) which reflects the accuracy of this multiparametric optimization approach.
- The robustness and effectiveness of this coupled approach underscore its potential as a valuable strategy for enhancing the quality and performance of manufactured specimens in diverse applications.

## Figures and Tables

**Table 1:** Factors and levels applied in the experimental work.

Factors	Levels			
	1	2	3	4
**SS [mm/s]**	70	90	110	130
**LH [mm]**	0.1	0.15	0.2	0.25
**PT [°C]**	190	200	210	220

**Table 2:** Sets of FDM process parameters applied to manufacture the initial specimens.

	Specimen															
	1	2	3	4	5	6	7	8	9	10	11	12	13	14	15	16
**SS**	70	70	70	70	90	90	90	90	110	110	110	110	130	130	130	130
**LH**	0.1	0.15	0.2	0.25	0.1	0.15	0.2	0.25	0.1	0.15	0.2	0.25	0.1	0.15	0.2	0.25
**PT**	190	200	210	220	200	190	220	210	210	220	190	200	220	210	200	190

**Table 3:** Experimentally assessed dimensions and porosity of the FDM-manufactured biocomposites.

Specimen	Dx [mm]	Dy [mm]	Dz [mm]	Porosity [%]
**1**	20.70	20.26	20.00	4.32
**2**	20.00	20.53	20.77	5.77
**3**	20.32	20.95	20.74	9.62
**4**	20.29	20.97	21.23	8.48
**5**	20.46	20.26	20.13	1.53
**6**	20.25	20.61	20.68	8.39
**7**	20.29	21.06	20.98	8.94
**8**	20.48	20.94	21.06	10.65
**9**	20.89	20.50	20.53	6.02
**10**	20.00	20.99	20.73	7.75
**11**	20.12	20.64	20.65	12.87
**12**	20.37	20.79	20.52	12.51
**13**	20.14	20.01	20.18	2.44
**14**	20.89	20.52	20.96	8.99
**15**	20.88	20.95	20.88	11.24
**16**	20.98	20.91	20.89	12.66

**Table 4:** Standardized values, Grey Relational Coefficient, Grey Relational Grades, and ranking of the experimental FDM-manufactured biocomposites.

Combination of FDM Process Parameters																
	1	2	3	4	5	6	7	8	9	10	11	12	13	14	15	16
**Standardized values**																
**Dx**	0.29	1.00	0.68	0.70	0.53	0.74	0.70	0.51	0.09	1.00	0.88	0.62	0.86	0.09	0.10	0.00
**Dy**	0.77	0.51	0.11	0.09	0.76	0.43	0.00	0.11	0.54	0.07	0.40	0.26	1.00	0.52	0.10	0.15
**Dz**	1.00	0.38	0.40	0.00	0.90	0.44	0.21	0.14	0.57	0.41	0.47	0.58	0.86	0.22	0.28	0.28
**Porosity**	0.75	0.63	0.29	0.39	1.00	0.40	0.35	0.20	0.60	0.45	0.00	0.03	0.92	0.34	0.14	0.02
**Grey Relational Coefficient**																
**Dx**	0.41	1.00	0.61	0.63	0.52	0.66	0.62	0.50	0.35	1.00	0.80	0.57	0.78	0.35	0.36	0.33
**Dy**	0.68	0.50	0.36	0.35	0.68	0.47	0.33	0.36	0.52	0.35	0.46	0.40	1.00	0.51	0.36	0.37
**Dz**	1.00	0.45	0.45	0.33	0.83	0.47	0.39	0.37	0.54	0.46	0.49	0.54	0.78	0.39	0.41	0.41
**Porosity**	0.67	0.57	0.41	0.45	1.00	0.45	0.43	0.38	0.56	0.48	0.33	0.34	0.86	0.43	0.37	0.34
**Grey Relational Grade**																
	0.69	0.63	0.46	0.44	0.76	0.51	0.44	0.40	0.49	0.57	0.52	0.46	0.85	0.42	0.37	0.36
**Ranking**																
	3	4	10	12	2	7	11	14	8	5	6	9	1	13	15	16

**Table 5:** Means of Grey Relational Grades assigned to all process parameters at each level.

Means of Grey Relational Grade			
	**Scanning speed**	**Layer height**	**Printing temperature**
**1**	**0.5553**	**0.699**	0.5217
**2**	0.5297	0.5338	0.5563
**3**	0.5115	0.4489	0.444
**4**	0.5031	0.4179	**0.5776**
**Δ(max − min)**	0.0522	0.2811	0.1336
**Rank**	3	1	2

**Table 6:** Results of ANOVA analysis for Grey Relational Grade.

Source	DF	Adj SS	Adj MS	F-Value	*p*-Value
**Scanning speed**	3	0.006417	0.002139	0.2	0.89
**Layer height**	3	0.190388	0.063463	6.05	0.03
**Printing temperature**	3	0.041268	0.013756	1.31	0.355
Error	6	0.062963	0.010494		
Total	15	0.301035			

**Table 7:** Means of x-dimensions assigned to all process parameters at each level.

Means of Dx [mm]			
	**Scanning speed**	**Layer height**	**Printing temperature**
**1**	**20.33**	20.55	20.51
**2**	20.37	**20.29**	20.43
**3**	20.35	20.4	20.64
**4**	20.72	20.53	**20.18**
**Δ(max − min)**	0.39	0.26	0.46
**Rank**	2	3	1

**Table 8:** ANOVA for x-dimension.

Source	DF	Adj SS	Adj MS	F-Value	*p*-Value
**Scanning speed**	3	0.4255	0.14184	1.48	0.311
**Layer height**	3	0.1803	0.06011	0.63	0.623
**Printing temperature**	3	0.4601	0.15338	1.6	0.285
Error	6	0.5738	0.09563		
Total	15	1.6398			

**Table 9:** Means of y-dimensions calculated for all process parameters at each level.

Means of Dy [mm]			
	**Scanning speed**	**Layer height**	**Printing temperature**
**1**	20.68	**20.26**	**20.61**
**2**	20.72	20.66	20.63
**3**	20.73	20.9	20.73
**4**	**20.6**	20.9	20.76
**Δ(max − min)**	0.13	0.64	0.15
**Rank**	3	1	2

**Table 10:** ANOVA for y-dimension.

Source	DF	Adj SS	Adj MS	F-Value	*p*-Value
**Scanning speed**	3	0.04287	0.01429	0.31	0.819
**Layer height**	3	1.10687	0.36896	7.98	0.016
**Printing temperature**	3	0.06457	0.02152	0.47	0.717
Error	6	0.27739	0.04623		
Total	15	1.49169			

**Table 11:** Means of z-dimensions calculated for all process parameters at each level.

Means of Dz [mm]			
	**Scanning speed**	**Layer height**	**Printing temperature**
**1**	20.68	**20.21**	**20.55**
**2**	20.71	20.79	20.57
**3**	**20.61**	20.81	20.82
**4**	20.73	20.93	20.78
**Δ(max − min)**	0.12	0.71	0.27
**Rank**	3	1	2

**Table 12:** ANOVA for z-dimension.

Source	DF	Adj SS	Adj MS	F-Value	*p*-Value
**Scanning speed**	3	0.03422	0.01141	0.25	0.861
**Layer height**	3	1.23787	0.41262	8.92	0.012
**Printing temperature**	3	0.22767	0.07589	1.64	0.277
Error	6	0.27759	0.04626		
Total	15	1.77734			

**Table 13:** Means of porosity calculated for all process parameters at each level.

Means of Porosity [%]			
	**Scanning speed**	**Layer height**	**Printing temperature**
**1**	**7.048**	**3.578**	9.56
**2**	7.377	7.725	7.762
**3**	9.787	10.668	8.82
**4**	8.832	11.075	**6.903**
**Δ(max − min)**	2.74	7.497	2.657
**Rank**	2	1	3

**Table 14:** ANOVA for porosity.

Source	DF	Adj SS	Adj MS	F-Value	*p*-Value
**Scanning speed**	3	19.64	6.5466	15.11	0.003
**Layer height**	3	143.729	47.9097	110.59	0
**Printing temperature**	3	16.376	5.4585	12.6	0.005
Error	6	2.599	0.4332		
Total	15	182.344			

**Table 15:** Experimentally assessed properties of the biocomposite cube manufactured with the optimized set of FDM process parameters.

Dx [mm]	Dy [mm]	Dz [mm]	Porosity [%]
20.115	20.556	20.220	1.673

**Table 16:** Standardized values, Grey Relational Coefficient, and Grey Relational Grade of the biocomposite cube manufactured with an optimized set of FDM process parameters.

Dx	Dy	Dz	Porosity
**Standardized values**			
0.883	0.482	0.822	0.988
**Grey Relational Coefficient**			
0.810	0.491	0.738	0.976
**Grey Relational Grade**			
0.754			

**Table 17:** Experimentally assessed x-, y-, z-dimensions and porosity of the cubes manufactured under the optimized process parameters for each optimization process.

SS [mm/s]	LH [mm]	PT [°C]	Dx [mm]	Dy [mm]	Dz [mm]	Porosity [%]
**70**	**0.15**	**210**	**20.1838**	20.3546	20.7324	6.37
**130**	**0.10**	**190**	20.0982	**20.0234**	20.4564	4.22
**110**	**0.10**	**190**	20.492	20.3526	**20.2976**	3.56
**70**	**0.10**	**220**	20.115	20.556	20.220	**1.673**

## Data Availability

Data presented in this article are available at request from the corresponding author.
